# Double-snare resection technique: a new approach for treating rectal carcinoid tumors

**DOI:** 10.1055/a-2318-2645

**Published:** 2024-06-05

**Authors:** Álvaro Rojas Sánchez, Fernando González-Panizo Tamargo, Carlos Carbonell-Blanco, Enrique Vazquez-Sequeros, Diego Juzgado Lucas

**Affiliations:** 1 Endoscopy Unit, Gastroenterology Department, University Hospital Quirónsalud, Madrid, Spain; 2 Endoscopy Unit, Gastroenterology Department, Vithas Aravaca Hospital, Madrid, Spain; 3 Department of Gastroenterology and Hepatology, University Hospital Gregorio Marañón, Madrid, Spain; 4 Division of Gastroenterology, University Hospital Ramon y Cajal, Madrid, Spain

Endoscopic resection is the preferred treatment for rectal carcinoid tumors (NETs), with different techniques described in the literature. Endoscopic mucosal resection (EMR) is feasible and is associated with a low risk of adverse events but a significant risk for incomplete resection 1 . Advanced endoscopy resection techniques have better success in achieving R0 resections but are associated with a higher risk of adverse events, are time consuming, and require highly skilled endoscopists to perform them 2 3 4 5 . We describe a new resection technique for rectal NETs <20 mm in diameter, the double-snare resection (DSR) technique, which may potentially overcome the aforementioned disadvantages.

We perform the DSR technique in three steps ( Video 1 ). First, a polypectomy snare (“resection snare”) is introduced through one of the instrument channels of the endoscope and opened in the intestinal lumen. Next, a second snare (“capture snare”) is introduced through the other channel and passed through the first snare ( Fig. 1 ). Then, the resection snare is partially closed and gently pulled back. Second, the capture snare is opened and used to take most of the lesion ( Fig. 2 **a** ), after which the resection snare is passed over the capture snare and used to encircle the whole lesion, ensuring a secure margin ( Fig. 2 **b** ). Third, we remove the lesion: the resection snare is closed, and the capture snare is opened and removed ( Fig. 3 ). The lesion is then resected using an electrosurgical generator, and the residual wound is closed with hemoclips.

**Fig. 1:**
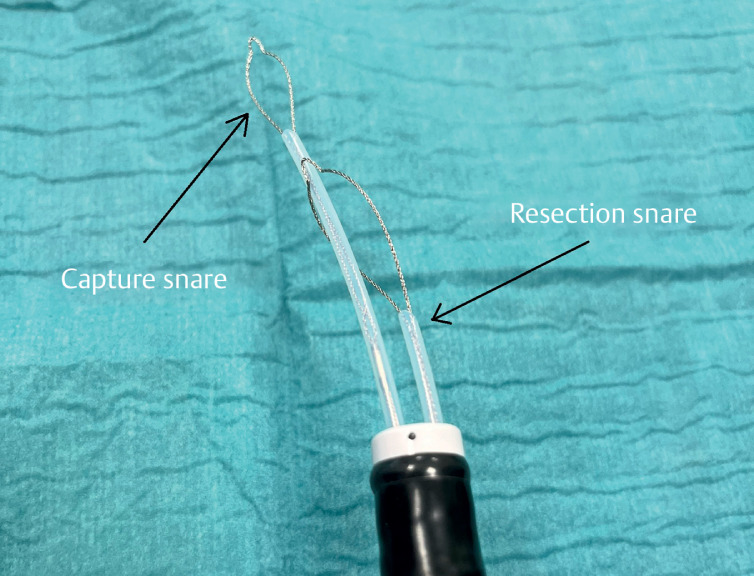
Resection snare and capture snare combination.

**Fig. 2:**
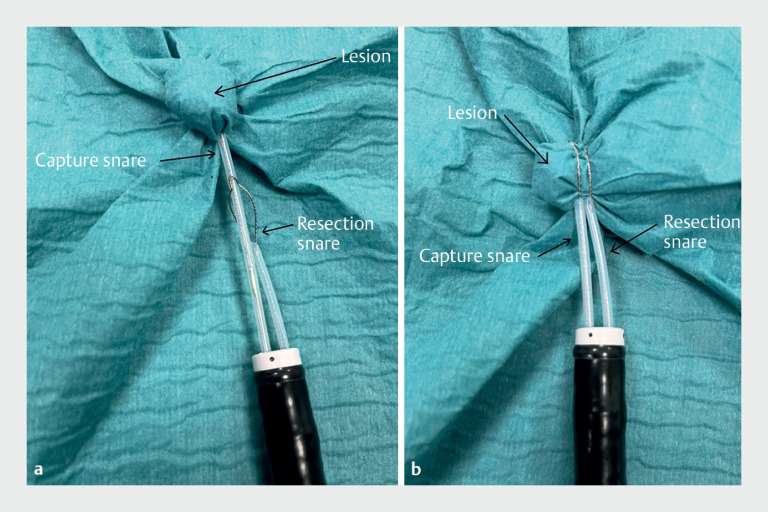
Positioning the snares over the lesion. **a** The capture snare takes the lesion. **b** The resection snare encircles the whole lesion.

**Fig. 3:**
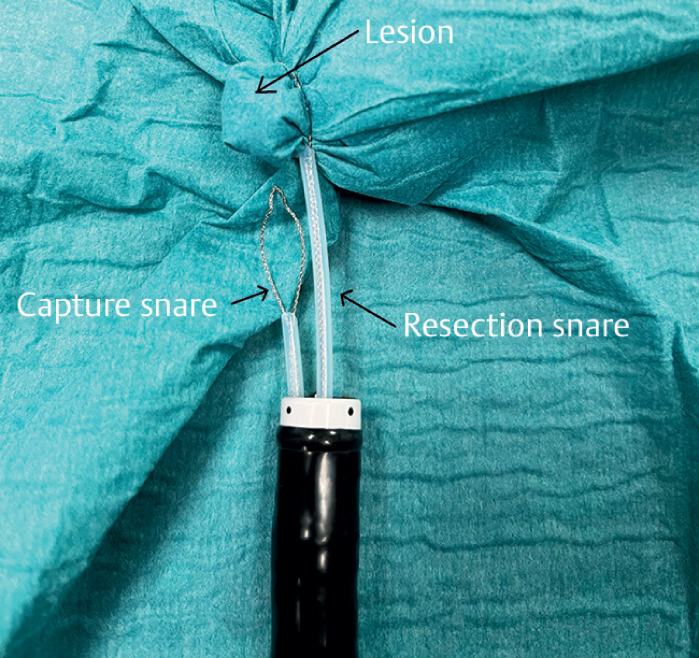
The capture snare is removed and the lesion is resected.

The double-snare resection procedure.Video 1

We consider that the DSR technique incorporates the advantages of all resection techniques described to date, with no inconveniences. It is easy to perform, faster than most advanced resection techniques, and can be achieved with just an endoscope and two standard polypectomy snares, which makes it a very cheap procedure. Finally, it is safe, with not a single adverse event in our experience, and allows high rates of R0 resection.

Endoscopy_UCTN_Code_TTT_1AQ_2AD

## Notes

Endoscopy E-Videos https://eref.thieme.de/e-videos
*E-Videos* is an open access online section of the journal *Endoscopy* , reporting on interesting cases and new techniques in gastroenterological endoscopy. All papers include a high-quality video and are published with a Creative Commons CC-BY license. Endoscopy E-Videos qualify for HINARI discounts and waivers and eligibility is automatically checked during the submission process. We grant 100% waivers to articles whose corresponding authors are based in Group A countries and 50% waivers to those who are based in Group B countries as classified by Research4Life (see: https://www.research4life.org/access/eligibility/ ). This section has its own submission website at https://mc.manuscriptcentral.com/e-videos .
